# Development, feasibility, and acceptability of SPoRT: a dating violence and sexual risk prevention intervention for college student-athletes

**DOI:** 10.1186/s40814-023-01413-z

**Published:** 2023-11-07

**Authors:** Nicole Jaffe, Meredith C. Jones, D. J. Angelone

**Affiliations:** 1 The Center for Psychology, 12499 Brantley Commons Ct #101, Fort Myers, FL 33907 USA; 2 Department of Psychology, Rowan University, 201 Mullica Hill Road, Glassboro, NJ 08028 USA

**Keywords:** Intervention, Development, Dating violence, Student-athletes

## Abstract

**Background:**

Student-athletes are one subgroup of college students in the USA at risk for dating violence and sexual risk behaviors. Despite this, research on student-athletes’ dating behaviors is limited; existing research pertains primarily to the National Collegiate Athletic Association (NCAA) Division I athletes and focuses on male student-athletes as perpetrators of dating and sexual violence. While some existing programs aim to reduce dating violence and promote healthy relationships, these programs are education based, and not tailored to the specific strengths and challenges of student-athletes. We therefore designed Supporting Prevention in Relationships for Teams (SPoRT), a novel, four-session prevention intervention for Division III student-athletes of all genders to reduce dating violence and sexual risk behavior by targeting knowledge and skills identified in pilot research, incorporating psychoeducation with techniques from cognitive-behavioral therapy, mindfulness, bystander intervention, and normative feedback.

**Methods:**

This study represents stage 1 of the National Institutes of Health (NIH) Stage Model for Behavioral Intervention Development, evaluating the feasibility and acceptability of SPoRT. We describe the development, content, and proposed delivery methods for SPoRT and evaluated the feasibility and acceptability of the program using a mixed-methods approach. Thirty college student-athletes (12 men, 18 women) completed questionnaires and participated in focus groups to provide feedback on the program’s length, timing, group size and dynamics, content, and suggestions for making the SPoRT prevention intervention more feasible and acceptable.

**Results:**

Our recruitment procedures were successful, and participants rated the program as feasible in terms of delivery methods and logistics. Participants liked that SPoRT was developed based on pilot data collected from student-athletes, brief, and skills based and tailored to athletic team needs. SPoRT was perceived as appropriate and relevant to student-athlete needs in terms of dating violence and sexual risk prevention knowledge and skills. Most participants (63%) rated the program as “excellent” and said they would recommend it to others.

**Conclusions:**

We found SPoRT to be both feasible and acceptable in terms of content and delivery. Suggested modifications will be incorporated into the SPoRT healthy relationships prevention intervention to be tested in an NIH Stage 1 efficacy trial.

## Key messages regarding feasibility

- What uncertainties about feasibility existed prior to this study?
  SPoRT is the first data-driven prevention intervention to promote healthy relationships and reduce dating violence and sexual risk behaviors among NCAA Division III college student-athletes of all genders in the USA. Because SPoRT is a novel program designed for a unique population, we needed to assess student-athletes’ perceptions of the program’s content, length, timing, group size, and composition, to determine the feasibility and acceptability of the prevention intervention before testing its efficacy in a randomized control trial. Consistent with the goal of designing this tailored prevention intervention with input from the community, it was important for us to collect qualitative data to assess whether participants found the program activities interesting and engaging and what changes they would like to see. An additional feasibility aim was to pilot our recruitment procedures to determine if we could successfully recruit student-athletes as participants, given their busy schedules.
- What are the key feasibility findings from this study?
  Using a mixed-methods approach, we evaluated student-athletes’ satisfaction with and perceptions of the proposed delivery methods and program content. Key feasibility findings include participants’ willingness to participate in SPoRT, satisfaction and positive evaluations of SPoRT’s content, length and timing of sessions, and the program being delivered within each team. Specific suggestions and modifications participants wanted to see in the program included more discussion of college hook-up culture and the inclusion of multiple activities to practice specific skills to reinforce learning. Our success with recruitment in the present study supports the feasibility of recruiting student-athletes to participate in a pilot randomized controlled trial.
- What are the implications of the feasibility findings on the main study’s design?
  These results will inform the next phase of the research, following the National Institutes of Health (NIH) Stage Model of Behavioral Intervention Development. Our findings indicate that we can proceed with a Stage 1 pilot randomized controlled trial of SPoRT with all student-athletes at one National Collegiate Athletic Association (NCAA) Division III university, which we will conduct after incorporating modifications to the program based on participant feedback.

## Background

Dating violence (DV) can be defined as the victimization or perpetration of physical violence, sexual violence, threats of physical or sexual violence, stalking, and psychological aggression against a partner in a dating relationship [1, 2]. A dating relationship includes a variety of behaviors, including spending time with a romantic interest with the expectation of future interactions, to a committed and exclusive partnership. Upwards of 47% of women and 38% of men first experience DV between the ages of 18 and 24 [1]. Further, DV is more common among college-aged couples relative to other age groups [3]. Among college students specifically, physical aggression occurs in 20 to 30% of dating relationships, while psychological aggression occurs in 50 to 80% of dating relationships, and sexual aggression occurs in 15 to 25% of dating relationships [4, 5].

Intercollegiate student-athletes are one group of college students at high risk for DV in the USA [6, 7]. The NCAA has three divisions: Division I athletic programs are the most competitive and award the most athletic scholarships, Division II programs are less competitive and award some scholarships, and Division III programs are less competitive than the other divisions and are not allowed to award athletic scholarships [8]. However, 40% of college student-athletes in the USA compete at the Division III level, with 438 college and university programs [9]. Notably, student-athletes are overrepresented as sexual violence perpetrators in judicial affairs complaints as compared to their non-athlete counterparts and are more likely to endorse acceptance of violence, rape myths, hostility towards women, and sexist beliefs than nonathletes [10–13]. Student-athletes also exhibit high rates of sexual risk behaviors (SRB), such as alcohol use before sex, condomless sex, and multiple sexual partners [14], which may lead to health outcomes such as unintended pregnancies and sexually transmitted infections [6, 11–13, 15–17]. Further, student-athletes report hazardous drinking, a known risk factor for DV and SRB involvement, with male student-athletes exhibiting high rates of alcohol use in conjunction with SRB [14, 18]. Specifically, alcohol use is associated with the perpetration of DV [19–23] among Division III student-athletes [14, 24, 25] and increases instances of unprotected sex [26].

In the USA, NCAA requires student-athletes to engage yearly in education on sexual violence prevention (NCAA.org, 2017). However, of the prevention programs targeting DV among college student-athletes, most are solely education based, and their efficacy has not been evaluated. Education, while necessary, is not sufficient for positive behavioral change [27] and may be unlikely to reduce rates of DV. Such programs include peer-led bystander training [28] and web-based format focused on alcohol use [29]. Instead, teaching evidence-based relationship skills in conjunction with psychoeducation may be more likely to elicit positive behavioral change [27]. Additionally, interventions are maximally effective when targeting unique strengths and challenges of any population [30].

*Supporting Prevention in Relationships for Teams* (SPoRT) is a prevention intervention developed to target the strengths and challenges of NCAA Division III student-athletes in establishing and maintaining healthy dating relationships. It is an inclusive, targeted, data- and skills-driven prevention intervention guided by the Centers for Disease Control and Prevention recommendations to the *White House Task Force to Protect Students from Sexual Violence* [27]. The overall goal of SPoRT is to have a positive impact on dating and relationship behaviors among Division III student-athletes by reducing risk for DV, SRB, and alcohol use through targeting several key mechanisms for change. These include psychoeducation about DV, SRB, alcohol use, and sexism; teaching cognitive-behavioral and mindfulness-based emotion regulation, stress management, and communication skills; and harnessing the strengths of the athletics and team environment to provide normative feedback and encourage bystander behaviors.

### Psychoeducation

Psychoeducation on DV in addition to SRB, alcohol, and sexism is a necessary start for successful behavioral change [27]. In addition, attitudes like hostile sexism and rape myth endorsement are associated with DV and SRB. This may be the result of sexism motivating perpetration or the endorsement of rape myths justifying perpetration [19, 31]. However, those attitudes are modifiable, and psychoeducation about rape myths, consent, and sexual risk reduction can reduce DV on college campuses [32]. Further, athletes with attitudes supportive of gender equity are less likely to report perpetrating DV [33]. Thus, these attitudinal risk factors are an important intervention target that may cultivate environments which are less conducive to DV and SRB [34].

### Emotion regulation and stress management skills

Targeting affective attitudes through emotion regulation and adaptive stress management strategies may increase positive outcomes as affective attitudes elicit behavioral change [35]. Further, emotion dysregulation is associated with maladaptive behaviors, such as alcohol-involved violence [36]. One stress management strategy commonly associated with emotion regulation is mindfulness. As an adaptive stress management strategy, mindfulness reduces stress [37, 38]. Specifically, among athletes, several facets of mindfulness, such as acting with awareness and non-judgement, are associated with stress reduction [39]. Mindfulness may also affect SRB, because it is associated with sexual consciousness and motivation [40].

Over the last decade, mindfulness-based interventions have also been designed to treat addictive behaviors, such as alcohol use [41]. Specifically, awareness of and reactions to aversive cognitive, affective, or physical states (i.e., cravings) are targeted through mindfulness-based interventions [42]. These include mindfulness-based relapse prevention [43, 44] and mindfulness-based substance abuse treatment for adolescents [45]. As such, it is reasonable to suggest that mindfulness may also help reduce alcohol use among college student-athletes.

### Communication skills

Another key mechanism for change includes increasing assertive communication skills. Dating partners can be taught to communicate effectively in order to establish and maintain healthy relationships. Assertive communication, which involves firm and direct verbal and nonverbal communication of one’s feelings, beliefs, and desires, may improve relationship quality and result in a reduction of SRB. Historically, intervention participants have been taught assertive communication to express a desire for safer sex behaviors [46, 47]. Interventions including a communication component have proven efficacious, resulting in more positive communication between dating partners [48, 49] and less DV [50]. Further, communication among college couples can increase safe sex behaviors, such as condom use [51, 52].

### Bystander behaviors

Bystander interventions can increase knowledge about DV and simultaneously lead to decreases in attitudes condoning of violent behaviors [53]. The intent of bystander interventions is to improve the decision-making process, during which bystanders notice a situation, address it, assess their own skills, and choose to intervene [54]. Among high school athletes, intention to intervene as a bystander is associated with less DV perpetration [33]. In addition, bystander interventions appear to reduce attitudinal risk over standard DV awareness education programs among college samples [55, 56]. Bystander interventions have also had a positive effect on attitudes towards DV, willingness to help, and other bystander behaviors [57–60]. Student-athletes often serve as leaders on campus and are in a unique position to address dangerous situations that may result in DV or SRB and intervene with their teammates and classmates.

### Normative feedback

Normative feedback corrects atypical assumptions influencing behaviors [61]. Providing normative feedback may decrease SRB, as young adults’ perceptions of their peers’ sexual activity, both frequency and quantity of partners, can be positively skewed; among student-athletes, unhealthy sexual behaviors are overestimated, leading to a false consensus effect [62]. The delivery of team-specific data can aid in the reduction of other SRBs, such as number of sex partners, frequency of sexual activity, and engaging safe sex behaviors prior to the onset of sexual activity. Normative feedback can also change perceived norms and reduce drinking behaviors among college students [61]. Further, online interventions for student-athletes utilizing normative feedback increase knowledge on DV behaviors and rape supportive beliefs [29]. As such, interventions should prioritize team-specific data-driven discussions in addition to evidence-based skills.

### The current study

Both phases 1 and 2 of this study represent Stage 1 of the NIH Stage Model for Behavioral Intervention Development [63]. Stage 1 includes modification to improve both the training materials and implementation of the new or revised intervention [64]. By adhering to the stage model of intervention development, we recognize that the scientific study of behavioral interventions neither begins nor ends with randomized control trials (RCTs). Instead, development begins with manual development (phase 1) and feasibility testing (phase 2).

The current study describes the development of the SPoRT prevention intervention manual and initial evaluation of the feasibility and acceptability of SPoRT. All study procedures were approved by the university’s institutional review board. The aims of a feasibility and acceptability study, as defined by the stage model, include demonstrating (a) participant acceptance of the new intervention, (b) the investigators’ ability to recruit from the target population, and (c) feasibility of intervention delivery [64]. This approach can be used to determine what aspects of the research methods and/or intervention protocol require modification [65]. Specifically, an evaluation of feasibility and acceptability is required in order to determine student-athletes’ satisfaction with the content and preferences for program delivery.

### Hypothesis

SPoRT differs from existing prevention interventions aimed to reduce DV and SRB because it is data-driven and targeted to the specific needs and strengths of NCAA Division III student-athletes. Further, developmental research with student-athletes informed SPoRT’s recruitment procedures, content, and delivery in order to increase feasibility. We developed SPoRT based on student-athletes’ needs and preferences to facilitate acceptability of the prevention intervention. As such, we predicted that NCAA Division III student-athletes would find SPoRT both feasible and acceptable.

## Methods

### Phase 1: SPoRT development

The intervention modules in SPoRT consist of psychoeducation about DV, SRB, and alcohol use risk reduction. challenging sexist attitudes and rape myths, and teaching brief emotion regulation, stress management, and communication skills [27, 57, 66–70]. Previous mixed-methods data collected from Division III student-athletes informed the inclusive development of SPoRT [24], accounting for diversity of gender and sexual orientation in addition to relationship experiences.

First, we conducted a survey of all student-athletes at the target Division III university. Quantitative data were collected from a sample of 350 Division III student-athletes (53.1% male, 45.4% female, 0.9% preferred not to say, 0.6% did not answer) from 16 sports teams (7 male teams and 9 female teams). These teams included football, men’s and women’s track and field, field hockey, men’s and women’s soccer, men’s and women’s swimming and diving, men’s and women’s cross country, baseball, men’s and women’s basketball, volleyball, softball, and women’s lacrosse. For a review of the quantitative data collected, see Table1.

**Table 1:** Phase 1 results

Themes	Description	Qualitative findings	Quantitative findings
Behavioral domains	Healthy and unhealthy behaviors relating to dating, sex, and relationships	Sexual assault, daring violence, alcohol use, social activities, relationship skills, intercourse, sexism, healthy and unhealthy relationships, bystander behaviors, social network, coping, sexual risk behaviors	• 56% experienced DV (*N* = 194) • 57% perpetrated DV (*N* = 197) • 17% did not obtain consent before sexual contact (*N* = 58)
Risk and protective factors	Various aspects of lifestyle specific to Rowan student-athletes that differentiates them from their non-athlete peers	Team culture, in season, out of season, specific sport, team strengths, team weaknesses, coaches, academic year, athletes vs. nonathletes	• 46% (*N* = 160) did not use a condom • 28% (*N* = 95) would not use a condom • 79% (*N* = 271) never HIV tested • 72% (*N* = 248) never tested for STDs • 62% (*N* = 215) hazardous alcohol use
Theory based	Potential intervention elements suggested by theoretical prevention models	Knowledge; skills; modeling; reinforcement; expectations; self-efficacy; bystander behaviors, attitudes, and efficacy; subjective norms; attitudes; intentions; perceived behavioral control; pluralistic ignorance; false consensus; impersonal sex; hostile masculinity; sexual aggression	
Intervention preferences	Preferences concerning intervention groups and delivery	Scheduling, facilitator, small groups, divided by gender, number of sessions, structure	

Quantitative data was only collected for behavioral domains and risk and protective factors

Next, we conducted focus groups to inform specific components of intervention delivery, such as when during the athletic season the intervention should take place, facilitator preferences, and size of intervention groups. Analysis of this data was guided by a consensual qualitative research (CQR) approach [71]. For a summary of core ideas, see Table 1. Both the qualitative and quantitative findings from the intervention development phase were used to inform the final SPoRT intervention manual used in this feasibility and acceptability study.

#### SPoRT content and delivery

The four 75-min SPoRT sessions addressed the following topics: (1) taking care of yourself and your team, (2) healthy relationships, (3) sexual violence, and (4) sexual risk. Each session was rehearsed first with research assistants to confirm the timing for each module within the session. See Table 2 for an outline of the content areas of each intervention module.

**Table 2:** SPoRT intervention modules

Session	Module title	Key mechanisms of change	Content
Session 1	Taking care of yourself and your team	Emotion regulation and adaptive coping strategies	How teammates can take care of one another, **emotion regulation**, coping and how substances influence coping, coping cards activity, **mindfulness**, **mindfulness activity**
Session 2	Healthy relationships	Communication skills	Healthy and unhealthy relationships, **sexual violence within dating relationships, sexual violence within dating relationships activity**, cycle of violence activity, safety cards activity, **communication skills**, **communication skills activity**
Session 3	Sexual violence	Attitudinal risk factors and bystander behaviors	Sexual violence, sexual violence activity, sexism and rape myths, **consent**, **did they get consent activity**, bystander interventions and identifying barriers
Session 4	Sexual risk	Sexual risk behaviors, alcohol and drug use	**Sexual risk**, sexual risk activity, **condom use**, **condom activity**, getting tested and talking about getting tested, alcohol use, alcohol use activity, review team goals and wrap-up

Content in bold was demonstrated in the phase 2 focus groups

SPoRT is designed to be delivered to one athletic team at a time, co-facilitated by a trained mental health clinician working with a student team leader, who is identified in consultation with team coaches and athletic staff. Evidence-based techniques facilitate group discussions and skills delivery. Normative feedback is derived from a baseline survey administered to teach team prior to session 1. Co-facilitators use a motivational interviewing approach [72] to deliver team-specific normative feedback data to build motivation for change. Cognitive-behavioral techniques [73] are used to teach, model, and reinforce skills. Additionally, mindfulness-based relaxation strategies are introduced to assist in targeting multiple key mechanisms for change by improving emotion regulation, reducing stress, increasing sexual awareness, and reducing rates of alcohol use.

### Phase 2: feasibility and acceptability of SPoRT

#### Participants

Eligible participants included intercollegiate student-athletes enrolled in a public NCAA Division III university in the northeastern USA with an undergraduate student population of approximately 15,000. All intercollegiate student-athletes over the age of 18 were eligible for participation and were randomly identified from team rosters and recruited via email. Of the 422 student-athletes invited, 71 responded. Of those who responded, 52 expressed interest in participating, 10 stated that they were not interested in participating, and 9 were lost to follow-up after requesting to learn more. An additional 22 were lost to follow-up after scheduling and failing to attend or expressing interest and failing to sign up for an available group. In total, 30 student-athletes participated in the focus groups: 12 male-identified and 18 female-identified. Of note, this response rate is consistent with student-athlete’s statements regarding their availability. For more information, please see intervention timing section of feasibility results.

#### Procedure

This study was approved by university’s institutional review board. Focus groups were held virtually via Webex video, and separated by gender, with male (*N* = 12) and female (*N* = 18) student-athletes. Groups were recorded, and student-athletes were prompted to not use any identifying information once the recording device was turned on. Any identifying information was removed during the transcription phase. Participants were compensated with US $20. The focus group facilitator (the first author) was a female doctoral student in clinical psychology with experience leading groups and 4 years of clinical training.

During the focus groups, the facilitator introduced each of SPoRT’s four sessions and provided an example of the intervention techniques to facilitate experiences. When reviewing the first session, *taking care of yourself and your team*, student-athletes discussed emotion regulation strategies and were taught mindfulness-based relaxation strategies through in vivo practice and encouraged to download a United States Veteran’s Affairs-sponsored mindfulness phone application. For the second session, *healthy relationships*, student-athletes learned the definition of DV, subsequently engaged in a DV activity, and learned assertive communication skills. When reviewing the third session, *sexual violence*, student-athletes discussed consent and watched a popular video explaining consent through sport metaphors. For the fourth session, *sexual risk*, student-athletes learned about SRB, reviewed a condom race activity, the impact of alcohol-use on SRB, and discussed a handout on SRB.

Following this presentation on some of the content, activities, and handouts included in SPoRT, student-athletes engaged in a semi-structured, guided discussion about their opinions on the acceptability and feasibility of the materials that were presented [74]. The focus group guide contained questions concerning (a) participant’s overall thoughts towards SPoRT, specifically what they liked and disliked; (b) preferences towards and appropriateness of interactive activities; (c) perception of the purpose of SPoRT and the ability to identify overarching domains and core ideas throughout intervention delivery; (d) specific skills embedded within the intervention; (e) what additional content should be included or subsequently, excluded from SPoRT; (f) acceptability of the discussions concerning difficult topics; and (g) when in their season student-athletes would like to receive SPoRT in addition to preferred length of the sessions. Follow-up probing questions were used to elicit complete, detailed responses, and after the guided discussion, a brief questionnaire was completed.

#### Quantitative approach

##### Measures

The feasibility and acceptability questionnaire contained 13 items. The items included were informed from a previous study examining the feasibility and acceptability of a DV and sexual risk intervention [75]. The first item concerns student-athletes’ willingness to discuss the topics presented in SPoRT, with student-athletes required to indicate whether they are willing to discuss these topics or not. Participants were then asked to describe their reasoning as to why they would or would not participate in SPoRT.

Student-athletes were then presented with seven Likert scale items asking about the acceptability of discussing their experiences or their teammates’ experiences with DV, safe sex behaviors such as condom use and discussing STIs, consent, dating relationships, and sexual encounters. Student-athletes were asked to indicate if it is very easy (1), easy (2), neutral (3), hard (4), or very hard (5) to address these topics. Two additional open-ended items queried whether there are any topics included in SPoRT that the athletes would like to see removed and if there were any topics athletes would like to see added.

##### Client satisfaction questionnaire (CSQ)

The CSQ [76] is an eight-item self-report measure of participant satisfaction. Designed to evaluate human service programs, the CSQ allows participants the opportunity to evaluate the services provided to them. We adapted the language of certain items in the CSQ to reflect the current study (i.e., replacing “program” and “service” with “intervention”). Each item contains four answer options, ranging in degree of satisfaction with the service or intervention received. For example, some answer options range from “almost all of my needs have been met” to “none of my needs have been met.” Scores of three or above reflect a positive evaluation, while scores of two or below reflect a negative evaluation. For example, answer options include the following: 4 = very satisfied, 3 = mostly satisfied, 2 = indifferent or mildly dissatisfied, and 1 = quite dissatisfied. The CSQ has strong internal consistency, with a Cronbach’s alpha of 0.92 [76]. For the current study, the CSQ demonstrated strong internal consistency, with Cronbach’s alpha of 0.86.

##### Qualitative approach

We transcribed focus group data via otter.ai and converted the transcripts into word processing documents. A research assistant reviewed these transcripts for fidelity, comparing them with the original focus group recordings. All identifying information was removed during this process. Coding was guided by a consensual qualitative research (CQR) approach, which allows for data to be collected through open-ended questions and consists of several coders throughout the analysis phase in order to foster multiple perspectives before a consensus is reached concerning the meaning of the data [77]. The research team, comprised of an auditor (the first author) and two undergraduate research assistants as coders, created a coding manual based on preliminary analysis of the transcripts and memos. Of note, coding focused on the primary constructs of interest: feasibility and acceptability. As such, while the NIH Stage Model for Behavioral Intervention Development framework guided the focus group agenda, an iterative process consistent with CQR guided data analysis. Research assistants entered transcript codes into spreadsheets. The coding manual was organized into domains, core ideas, categories, and sub-categories. This methodology is consistent with the three general steps of CQR, which are (1) divide data into domains, (2) construct core ideas within each domain, and (3) cross analyze the data to develop categories consistent with the core ideas within domains (71). The auditor reconciled disagreements across research assistants and cross-checked the research assistants’ coding with the transcripts.

## Results

### Quantitative results

Participants were encouraged to complete the CSQ given their knowledge of SPoRT following the overview of sessions and presentation on some of the content, activities, and handouts included in SPoRT. Participants expressed general satisfaction with SPoRT, with an average total score of 28 (*N* = 25, *SD* = 3) out of 32 on the CSQ, with higher scores expressing greater satisfaction. All eight items received mean scores of three or above, reflecting general satisfaction with SPoRT. Three items received scores of two or below, reflecting indifference or mild dissatisfaction. These findings are summarized in Table 3.

**Table 3:** Overview of client satisfaction questionnaire (CSQ)

Item	Excellent (4) % (*N)*	Good (3) % (*N)*	Fair (2) % (*N)*	Poor (1) % (*N)*	*M (SD)*
How would you rate the quality of the intervention you reviewed? (*N* = 25)	63.0 (17)	29.6 (8)	0.0 (0)	0.0 (0)	3.68 (0.48)
	Yes, definitely (4) % (N)	Yes, generally (3) % (N)	No, not really (2) % (N)	Definitely not (1) % (N)	*M (SD)*
Did you get the kind of intervention you wanted? (*N* = 26)	40.7 (11)	55.6 (15)	0.0 (0)	0.0 (0)	3.42 (0.50)
	Almost all of my needs have been met (4) % (N)	Most of my needs have been met (3) % (N)	Only a few of my needs have been met (2) % (N)	None of my needs has been met (1) % (N)	*M (SD)*
To what extent has our intervention met your needs? (*N* = 26)	55.6 (15)	40.7 (1)	0.0 (0)	0.0 (0)	3.48 (0.50)
	Yes, definitely (4) % (N)	Yes, I think so (3) % (N)	No, I don’t think so (2) % (N)	No, definitely not (1) % (N)	*M (SD)*
If a friend were in need of similar help, would you recommend our intervention to him/her? (*N* = 26)	63.0 (17)	33.0 (9)	0.0 (0)	0.0 (0)	3.65 (0.49)
	Very satisfied (4) % (N)	Mostly satisfied (3) % (*N)*	Indifferent or mildly dissatisfied (2) % (*N)*	Quite dissatisfied (1) % (*N)*	*M (SD)*
How satisfied are you with the amount of help you received? (*N* = 26)	63.0 (17)	29.6 (8)	3.7 (1)	0.0 (0)	3.62 (0.57)
	Yes, it helped a great deal (4) % (*N*)	Yes, it helped somewhat (3) % (N)	No, it didn’t really help (2) % (N)	No, it seemed to make things worse (1) % (N)	*M (SD)*
Has the intervention you reviewed helped you to deal more effectively with your problems? (*N* = 26)	29.6 (8)	55.6 (15)	11.1 (3)	3.7 (1)	0.3.19 (63)
	Very satisfied (4) % (N)	Mostly satisfied (3) % (N)	Indifferent or mildly dissatisfied (2) % (N)	Quite dissatisfied (1) % (N)	*M (SD)*
In the overall, general sense, how satisfied are you with the intervention you have reviewed? (*N* = 26)	51.9 (14)	44.4 (12)	0.0 (0)	0.0 (0)	3.54 (0.51)
	Yes, definitely (4) % (N)	Yes, I think so (3) % (N)	No, I don’t think so (2) % (N)	No, definitely not (1) % (N)	*M (SD)*
If you were to seek help again, would you come back to engage in this intervention? (*N* = 26)	51.9 (14)	40.7 (11)	3.7 (1)	0.0 (0)	3.50 (0.58)

In terms of intervention delivery, the majority (62%, *N* = 18) of participants noted a preference for receiving SPoRT across 4 weeks, with four 1-h and 15-min sessions occurring in the evenings. The majority of students (55%, *N* = 16) also indicated a preference for engaging in SPoRT during their freshman year. When asked about preferences towards the format of the activities embedded within SPoRT, 76% (*N* = 22) of participants identified a preference for games over videos (27.6%, *N* = 8), role-play activities (31%, *N* = 9), or audio recordings (3%, *N* = 10).

### Qualitative results

Following a CQR approach, domains and associated core ideas, categories, and sub-categories were developed and organized into a coding manual. Frequencies were not included as percentages, as CQR encourages utilizing labels to describe frequency. These labels include general, typical, and variant. “General” reflects a core idea, category, or sub-category included in all or all but one of the focus groups. “Typical” reflects a core idea, category, or sub-category included in more than half of the focus groups but less than all but one of the focus groups. “Variant” reflects a core idea, category, or sub-category included in at least two of the focus groups to the cutoff for typical. The label rare is used when a code idea, category, or sub-category is only included in one focus group.

### Feasibility

Core ideas concerning the feasibility of SPoRT included intervention length, intervention timing, and intervention group size. Within intervention length, length of sessions and number of sessions were included as categories, with attention and module length as sub-categories. Within intervention timing, categories included when in the year, time of day, day of the week, and individual schedules. Sub-categories for when in the year included preseason or camp, in-season, or out of season; sub-categories for time of day included mornings, afternoons, or evenings; and sub-categories for day of the week included weekdays or weekends. Within intervention group size, categories included small groups. Sub-categories for small groups included accessibility and comfortability. Those categories and sub-categories are described below, with examples.

#### Intervention length

Student-athletes noted that they found the intervention length, including length of sessions, and number of sessions, not only feasible but also a strength of SPoRT. Given the amount of content included and amount of time allotted between sessions (6 days, one session a week), four 1-h and 15-min sessions were deemed appropriate and according to one male student-athlete “very digestible.” Similarly, female student-athletes commented on the benefits of both the amount of and length of sessions:

I think it is also the fact that it’s over multiple days it’s not like the same time all at once is great because I think it’s creating a long-term narrative versus just I am here to sit here for 3 hours and have to just pay attention and then I leave.

Further, student-athletes also acknowledged that this structure allows for students to remain engaged in the content. Such a format also increases comfort with disclosure. For example, a male student-athlete noted the following:

Okay, so I think just being there four days, one day a week, I think it would build a bond between the team, especially with the same, the same people within the group.

When asked about the time allotted for activities and discussions, student-athletes responded positively. A female student-athlete stated the following:

I liked them, I felt like they were not over strenuous or invasive or overly time consuming. It really drove the points.

#### Intervention timing

When presented options for the timing of the intervention, student-athletes expressed a preference for either preseason or during the beginning of the athletic season. For example, a male student-athlete expressed the following:

Definitely preseason. When you are getting acclimated. If it is at a time when you are getting reacclimated, if something like this comes along, it can be very beneficial.

Participants also noted time constraints related to off-campus athletic competitions. In addition, they highlighted the need to consider first-year students, by making sure they receive the information included in SPoRT before becoming accustomed to the college atmosphere. Another male student-athlete stated the following:

I also think preseason for my group just because that’s when all the freshmen start to come in and you got to like, I guess, bring the message out early before seasons start so that it’s there.

Other preferences included engaging in SPoRT in the evenings during the week, as there are fewer classes in the evening, and the weekends are often reserved for competitions and other commitments. A female student-athlete noted her preference for the evening: “probably the evening because, like, a student-athlete schedule is packed.” Some student-athletes recommended replacing a practice session with SPoRT, as doing so would strengthen motivation to participate in SPoRT. One male student-athlete described the following:

I think if you can get into, like ending practice early and having a meeting people will be more inclined to pay attention, because I know whenever we have meetings after practice and we have just work our asses off and have work to do or meetings for club no one really wants to go into something they just see as mandatory session.

Across focus groups, student-athletes shared a preference for replacing or augmenting practice time with SPoRT due to their busy schedules.

#### Intervention group size

Smaller group sizes of up to eight to ten student-athletes provided student-athletes with an increased sense of comfort when discussing difficult topics, such as STIs and DV.

It's very small and since we are doing it with the same group each week, I feel like it’d be more comfortable environment to speak in.

Not only does a small group size foster a safe environment but it also contributes to an active learning environment where student-athletes can share their thoughts and experiences.

I just like the smaller better because it’s more in depth and I think creates a better environment and a better… speaking environment and trust within people as opposed to that one it's like here’s something we have to do and we're just going to get it over with.

Taken together, small group sizes were identified as a strength of SPoRT and the preferred format across focus groups.

### Acceptability

Core ideas related to the acceptability of SPoRT were group dynamics, intervention content, retention of intervention content, content to keep, suggested content, and content requiring modification. Categories embedded within group dynamics included gender, age and academic year, facilitators, and interaction styles. Sub-categories included cliques, taking the intervention seriously, planting seeds, and utilizing senior team leaders. Within intervention content, categories included the following: relatability, activities, interactive modules, discussion-based modules, gender-inclusive content, depth of content, healthy relationships, hook-up culture, and emotion regulation. Sub-categories of relatability specifically included talking to students and to student-athletes. Finally, categories of retention of intervention content included holding onto information and applying the information.

#### Group dynamics

Group dynamics were most prominently discussed in terms of age and academic year, in addition to interaction styles. Student-athletes noted a preference for diversity among SPoRT group members as it pertains to academic year in order to assist those younger team members, particularly freshmen, feel comfortable with their fellow team members. One female student-athletes explained as follows:

Maybe breaking senior cliques and freshman cliques and mixing them grade wise will help because people who are more mature about handling and opening up a little more than maybe like a freshman who’s maybe a little more immature.

Other group dynamics included interaction styles, which speaks to how group members feel most comfortable interacting with one another. For example, participants acknowledged that some group members may prefer interactive content and competition-based activities, while others may prefer watching videos and listening to discussions. As such, one male student-athlete suggested the following:

One idea for it maybe is have one, at the beginning, people might not be as comfortable with the other people there. So I mean a little bit less still interactive, but like a little bit less person to person until they get more comfortable. And later on, you could do ones that are more interactive with more of the people once they are more comfortable.

Student-athletes also described strategies that could help improve engagement in the group and session material. One such strategy includes involving a student team-leader as a co-facilitator, which participants found appealing. One male student-athlete explained several benefits for including student team leader as a co-facilitator:

I think having a team leader saying that guys let’s take this seriously will help to reinforce that because I think if it was just someone in an outside source trying to facilitate this it would not be taken seriously.

#### Intervention content

This category and its related sub-categories refer to student-athletes’ preference for specific modules and the content embedded within those modules. For example, content perceived favorably by student-athletes was relevant to student-athletes and their non-athlete counterparts. Other such preferences included interactive content (i.e., active discussions and competition-based activities), in-depth discussions, and information that is gender-inclusive in its presentation. For example, a male student-athlete spoke specifically to the activities included within SPoRT:

I like the activities. They were interactive. And that’s one thing I feel like with an activity we have to make it interactive. The less we have people pitch in the less they are gonna pay attention.

I think a lot of athletes learn from hands-on doing things. If you are using athletes, these are people who use their hands use their eye-hand coordination. They learn by doing most of the time.

This is in contrast to other interventions, which focus on lecture-based learning. The interactivity of SPoRT appeared appealing to student-athletes, as it increases participant’s attention and possibly engagement in the session material.

Consistent across focus groups, student-athletes discussed their enjoyment of the mindfulness exercise included in SPoRT. They also highlighted the benefits of the content on emotion regulation. A male student-athlete stated the following:

My personal favorite is just the breathing and emotional exercises. Sometimes when I am anxious it’s something I forget to do. I forget to stop and decompress. So, I just like taking a step back.

Two female student-athletes agreed, acknowledging the following:

I really like how the program started off, like when we talked about emotional management and detaching yourself from emotion and knowing that you are not your emotions.

The first one talked about mindfulness and more of your own emotions and regulating your emotions and that was not something I quite expected to be in it but I think it really important and is not talked about enough.

Other student-athletes identified the benefits of including additional content on hook-up culture and casual sexual relationships. A female student-athlete said:

I think maybe there should be a small section about hookup culture. Especially, college students see that a lot and like they might not know how to feel with it or go into it or feel pressured to go into something they are not comfortable with. But I think hookup culture is a big thing with college students.

As such, student-athletes spoke both of the content they identified as crucial to the goal of SPoRT—to teach student-athletes about healthy relationships—and content that is not yet included in SPoRT that may assist student-athletes in establishing and maintaining healthy relationships.

#### Retention

Student-athletes consistently noted the benefits of receiving and reviewing information primed for retention and able to be applied in everyday situations. For example, a male student-athlete described SPoRT as something “I wanted to pay attention because I felt it would be very useful for me to like, understand and know more about it.” Another benefit of SPoRT—the amount and length of sessions across 4 weeks—includes reinforcing session content between and during sessions. Student-athletes perceived this as beneficial for retention. This was compounded by the order of the session material, as noted by a female student-athlete: “I feel like the way you chose the order is like the best way like learn the information.”

#### Content to keep

Student-athletes identified several positive features of the SPoRT intervention content, including learning about and engaging in a mindfulness exercise, interactive and competition-based activities, a variety of activity formats, and consistent check-ins and group discussions. Further, student-athletes specifically compared the content and delivery of SPoRT to the content and delivery of other NCAA sanctioned interventions as described below:

…this kind of stuff it’s usually like, an hour-long meeting of just somebody like talking at you, and I feel like this can be an awesome way to like break it up, get involved and interact like not just sit down and stare at a PowerPoint and listen the whole time.

#### Suggestions

Some participants expressed interest in including additional information in SPoRT not already embedded within the modules. One such topic discussed frequently across focus groups was the casual hook-up culture of college. Other participants discussed creating multiple activities for one topic in an effort to increase engagement in the session material.

I just think it should be something where it’s individualized… because you know as people, we are very … some people lose track and stuff like that.

These changes or additions to the modules were coded as suggestions and reflect modifications that will be made to improve SPoRT for testing in an open pilot trial.

#### Modifications

Content that student-athletes identified as removable was identified as content subject to modification. For example, a female student-athlete discussed removing take-home activities designed to reinforce session content. She stated the following: “Honestly, I don't really like that part that much. It feels more like a class and a chore than a training.” Other modifications student-athletes discussed were regarding specific activities such as the consent and condom use activities in SPoRT. In discussing the condom use activity, a male student-athlete said:

The concept of having a relay race is cool in the aspect that it’s like everybody working together and trying to figure things like that and maybe there's a learning term for it but, tying back into what I was saying, like, that aspect of having a relay race might make it more of a joke than usual sexual interventions...I feel like the idea of the relay race will make it too informal if that make sense. Again, I would not know unless it started.

This student-athlete acknowledged that more interactive activities may be viewed as less serious than some of the other activities that focus exclusively on reinforcing SPoRT’s content without an interactive component. However, there was no consensus on material that should be removed across focus groups. Rather, student-athletes acknowledged their personal preference.

## Discussion

Developed in collaboration with Division III student-athletes, SPoRT represents an inclusive, targeted, data- and skills-driven intervention. SPoRT was designed to suit student-athletes’ needs and preferences. As such, student-athletes expressed satisfaction with SPoRT’s content in addition to the delivery of that content. This includes the activities and other modules within SPoRT, the number of sessions, the length of those sessions, and session group sizes. Our success recruiting student-athletes to participate in the present study also suggests that it is feasible to recruit student-athletes to participate in a randomized controlled trial of the prevention intervention to evaluate the efficacy of SPoRT.

Quantitative and qualitative data analyses revealed that student-athletes found SPoRT to be a feasible and acceptable way to promote healthy relationships among student-athletes. Quantitative results identified student-athlete’s comfort with discussing difficult topics included within SPoRT, such as DV, safe sex, and consent. These data also identified student-athletes’ willingness to participate in SPoRT and their preference for intervention delivery in the evenings, across 4 weeks, with four 1-h and 15-min sessions. Qualitative results revealed specific strengths of SPoRT, such as its appropriateness and relevance to student-athletes, interactive modules, order in which content is delivered, the variety of content (i.e., healthy relationship and safe sex behaviors), use of emotion regulation and mindfulness-based coping strategies, small group sizes, and senior team leaders as co-facilitators.

These results likely reflect the development of SPoRT as a collaboration between researchers and student-athletes. While these data continue to contribute to our understanding of our target population, they also reflect some necessary changes to SPoRT. These changes including allowing flexibility in the activities are included within the session modules and the addition of content that speaks to casual relationships or hook-ups. This can be done through adding alternative activities based on athletes’ engagement in SPoRT and embedding content that describes student-athlete hook-up culture.

Making the proposed changes identified across focus groups can increase student-athletes’ satisfaction with SPoRT and improve outcomes. For example, including alternative activities allows for our facilitators to utilize those activities best suited to the group. Hands-on or physically oriented learners can engage in more active activities, while verbal or visual learners can take part in other activities that speak to both their learning style and strengths. This is consistent with previous literature stating student groups vary in learning style [78, 79]. Further, hook-up culture is an important topic to include within SPoRT. As such, by including discussions concerning risk factors associated with hook-up culture, we will increase the relevance and relatability of SPoRT. For example, hook-up culture can be used to describe SRB and the subsequent importance of practicing safe sex strategies in an attempt to reduce risk for STIs and unintended pregnancy.

Limitations of this study include how the structure and content of SPoRT was presented to student-athletes. Rather than engage participants in SPoRT in full intervention, participants received an overview of SPoRT while engaging in select discussions and activities. As such, these data do not reflect student-athletes’ perception of the full intervention. While this was done intentionally given certain constraints as the result of COVID-19, it is possible that intervention trial results may differ based on student-athletes’ ability to engage in SPORT as intended, in four 1-h and 15-min sessions across 4 weeks.

Future directions should include analyzing the preliminary efficacy of SPoRT following an open pilot trial of the full SPoRT intervention. This is consistent with the NIH Stage Model for Behavioral Intervention Development [63]. Identifying preliminary efficacy through an open pilot trial is included within Stage 1 and answers the question “does it work?”. Following completion of an open pilot trial, Stage II consists of randomized clinical trials to evaluate the efficacy of a manualized and pilot-tested intervention [64]. More than one RCT is often included within Stage II, as Stage III involves generalizability to a larger sample and implementation concerns, in addition to cost-effectiveness and marketing issues [64].

Other future directions involve identifying the generalizability of SPoRT. While SPoRT was designed to target the needs and behaviors of Division III student-athletes, future studies can assess the generalizability of SPoRT to all NCAA divisions. Identifying specific differences between Divisions I, II, and III student-athletes can inform changes needed to modify SPoRT to target either NCAA Division I, II, or III student-athletes at a variety of universities. As such, our future goal is to understand how behaviors, needs, and preferences differ across Division I, Division II, and Division III student-athletes. Ultimately, we hope that SPoRT can meet the healthy relationship prevention intervention needs of student-athletes across divisions.

## Data Availability

The datasets generated from the current study are available from the corresponding author upon reasonable request.

## Abbreviations

SPoRT: Supporting Prevention in Relationships for Teams
NCAA: National Collegiate Athletic Association
DV: Dating violence
SRB: Sexual risk behaviors
NIH: National Institutes of Health
CQR: Consensual qualitative research
CSQ: Client Satisfaction Questionnaire
